# A two‐step treatment planning strategy incorporating knowledge‐based planning for head‐and‐neck radiotherapy

**DOI:** 10.1002/acm2.13939

**Published:** 2023-02-24

**Authors:** Han Liu, Benjamin Sintay, David Wiant

**Affiliations:** ^1^ Department of Radiation Oncology Cone Health Cancer Center Greensboro North Carolina USA

**Keywords:** knowledge‐based planning, optimization method

## Abstract

**Purpose:**

There has been much research interest in automated head‐and‐neck (HN) planning with the goal of reducing planning time and inter‐planner variability while improving plan quality. However, clinical uses are still limited and institution‐dependent due to the plan complexity. This work aims to investigate whether the use of a novel semi‐automated two‐step optimization method (TSP) can improve the quality and efficiency of planning while providing a simple framework that other institutions can follow.

**Methods and materials:**

Forty patients (two and three prescription isodose levels) were retrospectively studied. Plans were generated by TSP which incorporates a knowledge‐based planning solution. Comparisons were performed for plan conformity and selected dose‐volume indices between clinical plan (CP) and TSP. Blind reviews were carried out by 15 clinicians to determine preference between the CP and TSP, as well as clinical suitability.

**Results:**

For majority of patients studied, TSP had similar or slightly better conformity for the high‐dose PTV, and better conformity for the low‐dose PTV and 45 Gy isodose lines compared to CP. The only statistically significant difference observed for the serial organs was a reduction of the spinal cord maximum dose with TSP. Except for left parotid gland (*D*_mean_ and *V*_30_ for both 2R× and 3R× groups) and oral cavity (*D*_mean_ for 3R× group), TSP had significant dose reductions for all parallel organs compared to CP. Blind reviewers either showed preference/no preference for 57.2%/21.7% (2R×) and 57.5%/27.8% (3R×) of TSP compared with CP. Excluding no preference votes, 60% of TSP were preferred. TSP was selected majority of the time when looking at the vote distribution for each patient individually.

**Conclusion:**

Our TSP allows plans to be created within 90‐min time frame while offering improvements in plan quality and less inter‐planner variability as compared to traditional planning techniques.

## 1 INTRODUCTION

Radiotherapy is employed as a primary treatment option or as an adjuvant to surgery for patients with head‐and‐neck (HN) cancer. The management of quality of life after HN radiotherapy is very challenging, depending on the site of the cancer, the stage and extent of the disease.^1^, ^2^ An important goal of radiotherapy treatment planning is to achieve adequate target coverage while sparing the surrounding organs‐at‐risk (OAR) and healthy tissues as much as possible to help reduce toxicity.

In the last two decades, intensity modulated radiotherapy (IMRT) and volumetric modulated arc therapy (VMAT) have become standard planning techniques in HN radiotherapy, due to their ability to give higher doses to the target while still provide superior dose conformity (less radiation‐induced toxicity) than the traditional 3D‐conformal radiotherapy (3D‐CRT) technique. Currently, VMAT has become more and more popular in practice since its first implementation into the clinic.^3^, ^4^ Compared to IMRT, VMAT has the advantage of decreasing treatment delivery time, thus reducing patient discomfort and the risk of intrafraction motion.^5^, ^6^, ^7^ Although these advanced IMRT and VMAT techniques can achieving highly conformal dose distributions and sharp dose fall‐off around the target, HN planning remains very challenging due to the location and size of the target (gross and microscopic diseases, lymph node) and the presence of more than 30 critical structures. Since targets to be covered and critical organs to be spared for a new HN patient must be carefully customized, traditional HN planning techniques often involve a trial‐and‐error that is sometimes very time‐consuming depending on the patient's anatomy. And although a standard set of dose constraints for critical organs have been suggested by clinical trials,^8^, ^9^, ^10^ it is often not possible to meet these guidelines in practice due to the proximity of the OAR to the target. This forces the planner to make difficult decisions on how to balance target coverage and OAR sparing, which can lead to dependence on the skill level of the planner and to a great deal of inter‐planner variability. Furthermore, the final approved plan for patient treatment is not always optimal and subject to the planner's experience and skill, and may be institution‐dependent from a variety of factors.^11^, ^12^

Recently, several automated solutions have been commercialized for the HN planning process with the intention of reducing planning time while producing consistent and clinically acceptable treatment plans. One such approach is known as RapidPlan™ (Varian Medical Systems, Palo Alto, USA) used in conjunction with the Eclipse treatment planning system (TPS).^13^, ^14^ RapidPlan is a knowledge‐based planning (KBP) software that generates estimated dose‐volume histograms (DVHs) for photon optimization based on previous patient anatomy and dose distributions. By leveraging dosimetric and geometric information from previous clinical plans (CP), KBP has the capability to reduce planning time and inter‐planner variations. Another strategy is the auto‐planning module of the Pinnacle^3^ TPS (Philips Medical Systems, Fitchburg, WI) that uses a template‐based optimization algorithm to mimic the manual iterative planning process.^15^, ^16^, ^17^ Besides the commercially available software mentioned above, tremendous efforts have been made to develop automated solutions for different treatment sites on a variety of TPS platforms.^14^, ^18^ The adaption of plan automation in a simple case with a single PTV (e.g., prostate) has been shown to be beneficial in improving plan quality and planning efficiency,^19^ however the application in HN (usually multiple prescription levels) is still limited and institution dependent. The purpose of this study is to investigate whether the use of a novel two‐step optimization method (TSP) (semi‐automated) can improve plan quality and efficiency of HN treatment planning, while reduce the inter‐planner variability.

## 2 METHODS AND MATERIALS

### 2.1 Patient data and characteristics

Forty HN cancer patients with various tumor sites and stages treated at our institution (two different facilities) were retrospectively studied. All patients underwent a planning computed tomography (CT) scan on Philips Brilliance Big Bore CT scanner (Philips, Cleveland, OH) with 2 mm slice thickness. Two different prescriptions were used in this study: (1) three prescription dose levels (3R×): a total dose of 70 Gy over 35 fractions to the primary tumor, and a simultaneous integrated boost (SIB) of 63 Gy to regions with microscopic disease and 56 Gy to the low‐risk nodal region; (2) two prescription dose levels (2R×): a total dose of 70 Gy over 35 fractions to the primary tumor, and a SIB of 54 Gy to the microscopic disease and low‐risk nodal region. Table 1 lists the tumor locations and characteristics for all patients used in this study.

**TABLE 1:** Patient characteristics

Patient # (3R×)	Tumor site	*V*_PTV70_ (cc)	*V*_PTV63_ (cc)	*V*_PTV56_ (cc)	Patient # (2R×)	Tumor site	*V*_PTV70_ (cc)	*V*_PTV54_ (cc)
1	Tongue	24.6	199.3	492.8	1	Tongue	86.3	591.5
2	Oropharynx	177.3	407.3	146.3	2	Laryngopharynx	70.6	274.2
3	Oropharynx	243.9	243.7	364.4	3	Tonsil	67.4	527.3
4	Oropharynx	113.8	151	374.7	4	Parotid	25.7	254.4
5	Oropharynx	225.2	269.9	495	5	Tonsil	96.4	397.3
6	Laryngopharynx	275.8	499.7	207.6	6	Laryngopharynx	39.7	367.3
7	Oropharynx	233.8	333.2	583.7	7	Nasopharynx	27	297.4
8	Laryngopharynx	318.6	475.7	220.8	8	Tongue	115.4	420.8
9	Nasopharynx	30.9	189.7	334.7	9	Tonsil	67.4	299.1
10	Laryngopharynx	243	539.7	317.1	10	Tongue	145.2	649.9
11	Larynx	106	178.4	432	11	Tonsil	55	426.8
12	Tonsil	256.4	532.3	380.9	12	Tonsil	267.9	691.1
13	Tonsil	217.6	668.9	258	13	Tonsil	22.3	231.5
14	Tonsil	237.6	406.1	313.9	14	Larynx	56.9	491.9
15	Larynx	137.3	326.1	373.6	15	Tongue	84.6	479.5
16	Larynx	56.9	111.4	301.4	16	Laryngopharynx	70.6	274.2
17	Larynx	80.8	165.3	388.6	17	Pharynx	45.4	320.3
18	Larynx	108.1	129.5	302.3	18	Tongue	86.3	591.5
19	Tonsil	162.1	176.8	438.7	19	Parotid	27.3	149.3
20	Larynx	38.3	82.9	319	20	Tonsil	65.3	507.3

### 2.2 Treatment planning

#### 2.2.1 Clinical plan

All CP were optimized in Eclipse version V13/V15 with the photon optimization (PO) algorithm. The primary goal of treatment planning was to deliver 100% of the prescribed doses to at least 95% of the volume for each individual PTVs while sparing the critical organs and normal tissues as much as possible. The planning guidelines for OAR and unspecified normal structures are based on Radiation Therapy Oncology Group (RTOG) studies RTOG‐0615,^8^ RTOG‐0920, RTOG‐0022^9^ and QUANTEC (quantitative analysis of normal tissue effects in the clinic),^10^ with adjusted planning goals based on each individual patient's specific anatomy. All CP were generated with two or three 6 MV VMAT beams and treated on the Varian TrueBeam platform.

#### 2.2.2 Two‐step optimization method

A new two‐step optimization algorithm has been developed for the HN planning in this study.

##### 2.2.2.1 Step 1: Optimize plan with KBP tool

A RapidPlan™ model was trained with a set of 90 HN patients previously treated at our clinic (planned with TomoTherapy or Eclipse TPS) with screening to exclude special circumstances such as nonstandard anatomy or abnormal dose criteria. Outliers were detected using a vendor‐provided model analytics tool. Each case in the RapidPlan model library was analyzed individually to evaluate whether removal from the cohort was appropriate. Due to the large variabilities of the anatomic relationship between the PTV and OARs in HN planning, we decided to use the following un‐involved structures (subtract the high‐dose PTV from the involved structures) in the model building rather than the involved ones: larynx, pharynx, oral cavity, left and right parotid glands. A posterior avoidance structure (posterior expansion of the brainstem and spinal cord contours) to help normal tissue sparing was also built into the RapidPlan model. Instead of aiming for adequate target coverage, our main focus at step 1 is to obtain reasonable OAR and normal tissue sparing, which is achieved by using the RapidPlan model.

##### 2.2.2.2 Step 2: Manual plan improvement

Upon the completion of step 1 optimization, a second round of optimization was initialized with the goal of achieving appropriate PTV coverage of the prescription dose, control the hot spots, as well as improve the plan conformity with the help of ring structures. At step 2, dose constraints and weighting were interactively adjusted to achieve the clinical guideline for the following four serial organs: mandible, spinal cord, brachial plexus and brainstem. At this stage, we continued the optimization with the step 1 result, then returned to the multi‐resolution (MR) level 3 immediately after the optimization initialized.

### 2.3 Dosimetric evaluation

Plan quality was evaluated by comparing plan conformity and selected dose‐volume indices between the CP and TSP. All plans were normalized such that at least 95% of the PTVs received the prescription doses. To have a fair comparison, the high‐dose PTV (70 Gy) coverage of the TSP was forced to be identical to the CP for the same patient. The maximum dose (*D*_max_), minimum dose (*D*_min_) and target homogeneity were compared for all PTVs between the CP and TSP. In this study the PTV homogeneity index was defined as:

$$HI=\frac{D_{2\%}}{D_{98\%}}\;,$$

where D_2%_ and D_98%_ are doses to 2% and 98% of the PTV volume respectively. Dosimetric parameters were compared for critical organs, including *D*_0.1cc_ (dose to 0.1cc of the volume, a representative of maximum dose) for serial organs (mandible, brainstem, spinal cord and brachial plexus); mean dose for parallel organs: larynx, pharynx, oral cavity, left and right parotids, esophagus; *V*_30Gy_ (volume receiving 30 Gy and higher) for left and right parotid glands, *V*_50Gy_ for pharynx; and *D*_0.1cc_ and mean dose for all other OARs such as optical structures and cochlea. The paired Student *t*‐test was used to evaluate the statistical significance of different dosimetric indices between the CP and TSP.

The evaluation of plan conformity between the CP and TSP was assessed by the following ratio:

$$R=\frac{V_{IDL}\;\left(\mathrm{TSP}\right)}{V_{IDL}\;\left(\mathrm{CP}\right)\;},$$

where *V_IDL_* is the volume encompassed by selected isodose lines (IDLs). In this study the IDLs were chosen to be 70 Gy, 54 and 45 Gy for 2R× patient group; and 70 Gy, 63 Gy, 56 and 45 Gy for 3R× patient group. The ratio *R* defined above is an adequate representation of plan conformity comparison between the CP and TSP since the target coverage was forced to be same. *R* < 1 indicate the TSP has better conformity than the CP, and vice versa.

### 2.4 Blind review

For each pair of CP and TSP, blind reviews were carried out for all 40 patients by a group of 15 clinicians (physicists, dosimetrists and radiation oncologists) to determine the preference between them, as well as the clinical suitability of each TSP and CP plan. By direct comparison of the isodose distribution, target coverage, location and degree of hot spots, OAR/normal tissue sparing, and plan conformity, clinicians selected a preference from two anonymized plans for each patient, with the option of no preference. Clinicians were also asked to provide the feedback whether the TSP and CP plans were clinical acceptable. A comment section was provided to gain additional insight. The purpose of the blind review approach was to assess the performance of the new two‐step optimization algorithm beyond looking at only one‐dimensional dosimetric guidelines.

## 3 RESULTS

**FIGURE 1:**
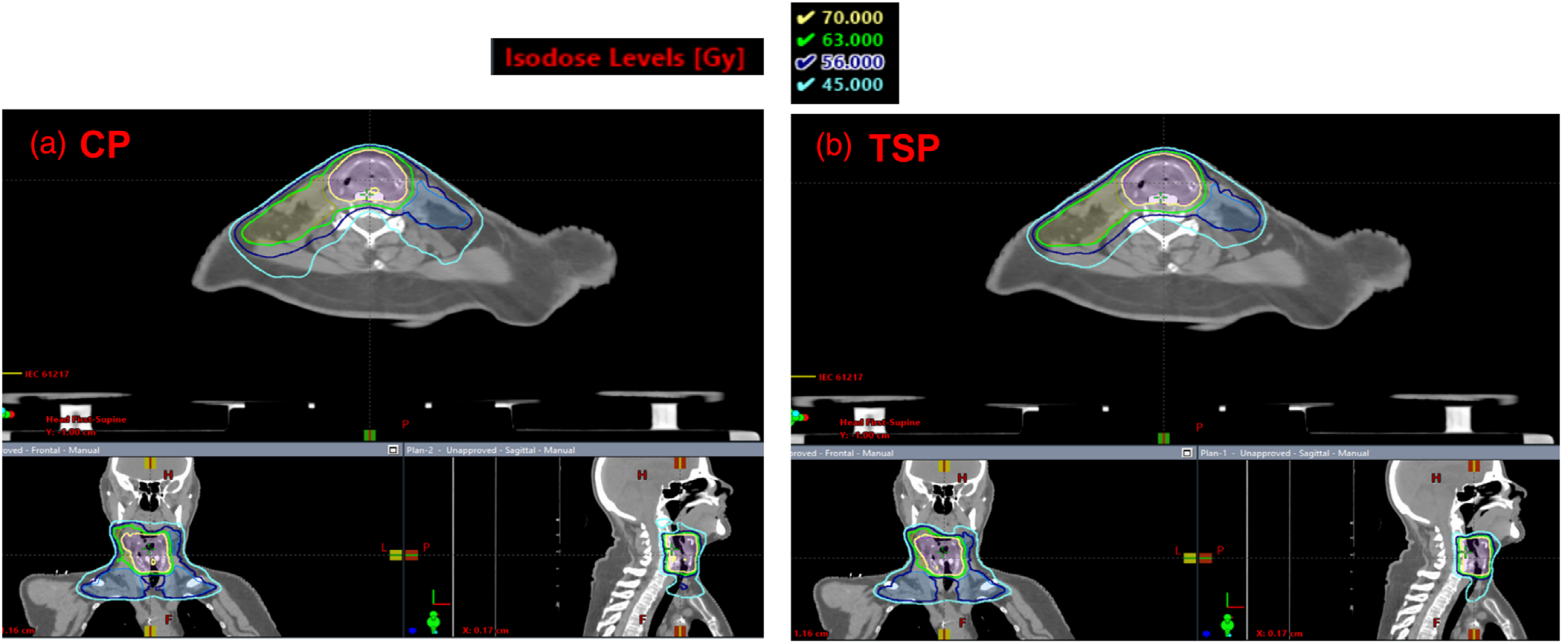
Comparison of isodose distributions between the CP (left) and TSP (right) for a representative 3R× HN patient on the axial, coronal and sagittal planes. Isodose lines are shown for three prescription dose levels (70 Gy, 63 Gy, 56 Gy) and 45 Gy

Figure 1 compares the isodose distributions between the CP (left) and the TSP (right) on axial, coronal and sagittal planes for a representative 3R× patient. The isodose lines shown are 70 Gy (yellow), 63 Gy (green); 56 Gy (blue) and 45 Gy (cyan). All three prescription isodose lines were comparable between the CP and TSP, while the TSP had better conformity in 45 Gy isodose line compared to the CP, indicating better sparing of the normal tissue around the target region.

**FIGURE 2:**
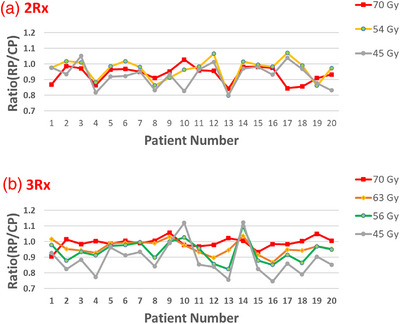
Plan conformity comparison between the CP and TSP. (a) 2R× patient group; (b) 3R× patient group. Ratio < 1 indicates that the TSP has better conformity than the CP for that specific isodose line

Figure 2 plots the ratio of volume encompassed by selected IDLs between the TSP and CP. For majority of patients studies, the TSP had slightly better or similar conformity for the high‐dose PTV and better conformity for the low‐dose PTVs and 45 Gy isodose lines compared to the CP.

**FIGURE 3:**
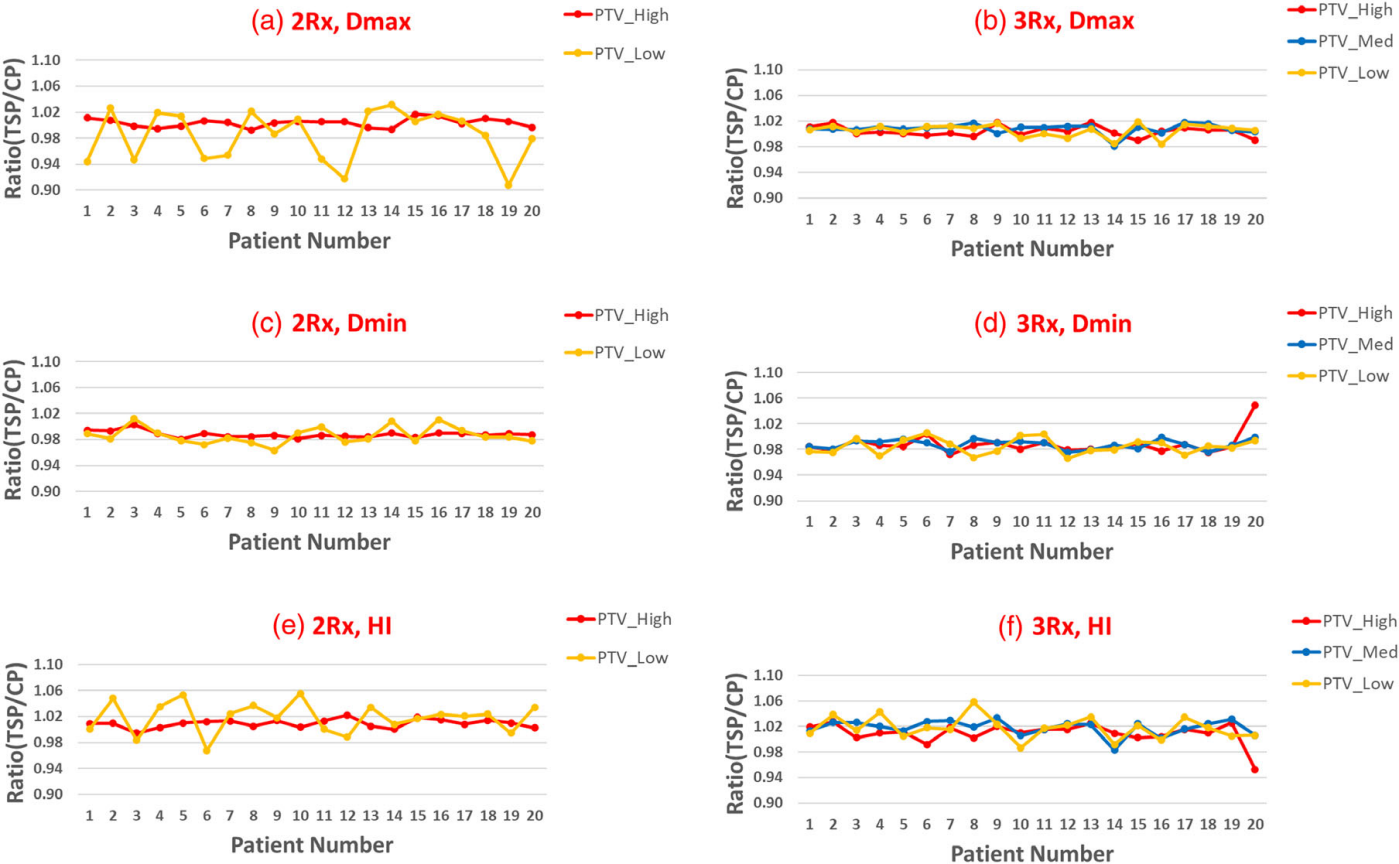
Dosimtric (maximum dose, minimum dose and homogeneity index) comparisons for PTVs between the CP and TSP. (a,c,e) 2R× patient group; (b,d,f) 3R× patient group

The ratios of maximum dose, minimum dose and homogeneity index for all PTVs between the TSP and CP are plotted in Figure 3 for both 2R× and 3R× patient groups. For 3Rx patient group, no significant changes in *D*_max_, *D*_min_ and *HI* were observed for all PTVs between the CP and TSP for majority of patients studied. For 2R× patient group, comparable dosimetric indices (within 2%) were observed for high‐dose PTV, as well as *D*_min_ for low‐dose PTV. About 1/3 of the studied patients showed some fluctuations of *D*_max_ and *HI* between the CP and TSP.

**FIGURE 4:**
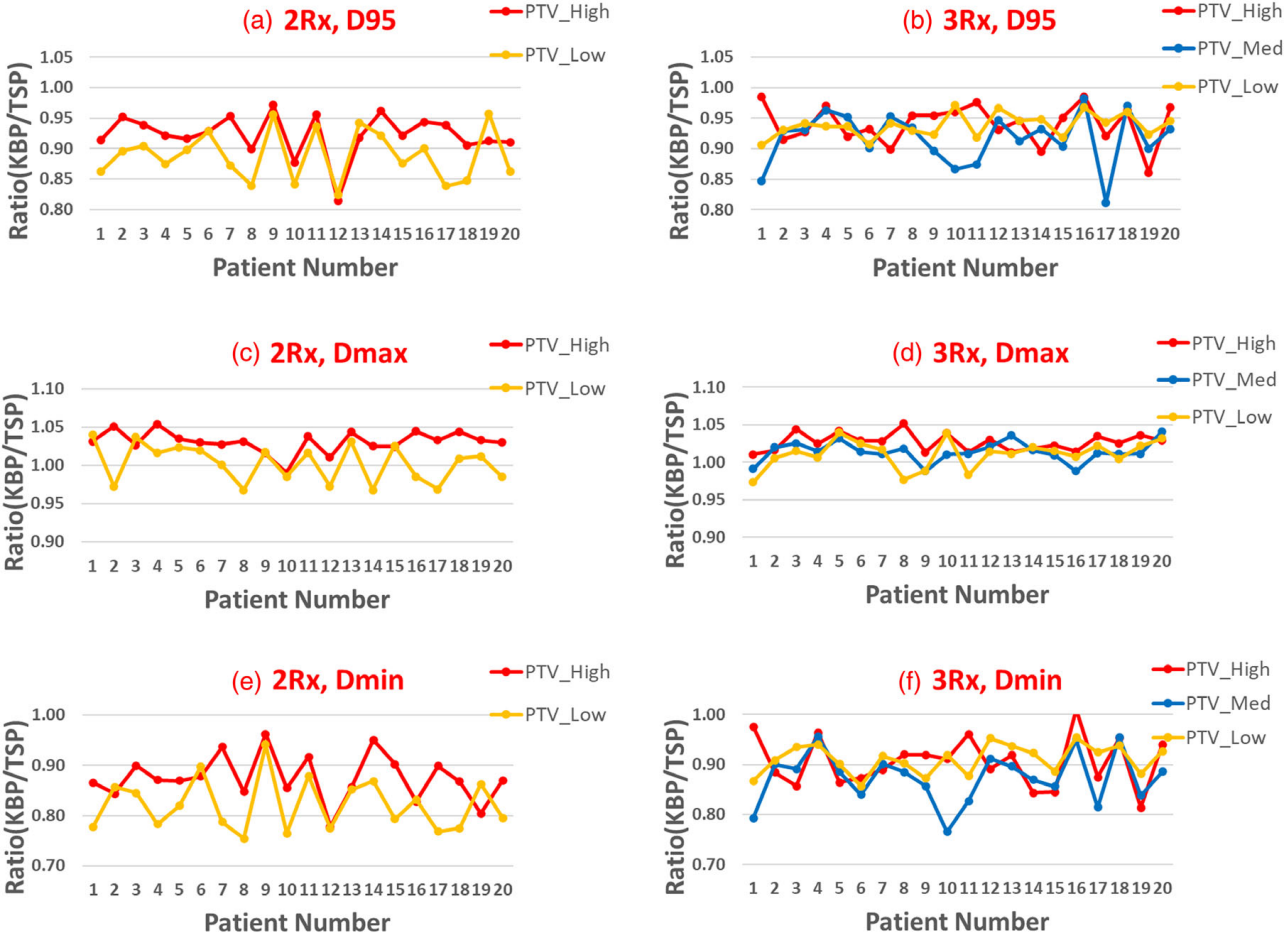
Dosimtric comparison (*D*_95_, maximum dose and minimum dose) comparisons for PTVs between the KBP (step 1) and TSP (step 2). (a,c,e) 2R× patient group; (b,d,f) 3R× patient group

The ratios of *D*_95_, *D*_max_, and *D*_min_ for PTVs between the KBP (step 1) and TSP (step 2) are plotted in Figure 4. Compared to the TSP, the KBP has worse target coverages to all PTVs for both 2R× and 3R× patient groups. This can be seen by the fact that the *D*_95_ and *D*_min_ ratios of the KBP and TSP are less than 1, with a mean *D*_95_ ratio of 0.92 ± 0.03 (PTV_High), 0.89 ± 0.04 (PTV_Low) for 2Rx patient group, and 0.92 ± 0.03 (PTV_High), 0.92 ± 0.04 (PTV_Med), 0.94 ± 0.02 (PTV_Low) for 3Rx patient group. The mean *D*_min_ ratio are 0.88 ± 0.05 (PTV_High), 0.82 ± 0.05 (PTV_Low) for 2Rx patient group, and 0.91 ± 0.05 (PTV_High), 0.87 ± 0.05 (PTV_Med), 0.91 ± 0.03 (PTV_Low) for 3Rx patient group. Furthermore, plans from the KBP have higher *D*_max_ than those from the TSP, with a mean *D*_max_ ratio of 1.03 ± 0.01 (PTV_High), 1.00 ± 0.02 (PTV_Low) for 2Rx patient group, and 1.03 ± 0.01 (PTV_High), 1.01 ± 0.01 (PTV_Med), 1.01 ± 0.02 (PTV_Low) for 3R× patient group. This is not surprising due to the fact that the main focus at step 1 is to obtain reasonable OAR and normal tissue sparing rather than adequate target coverage.

The dose differences (*D*_max_) between the CP and TSP were within 1 Gy for all the optical structures (left and right eyes, left and right lens, left and right optical nerves and optical chiasm). For 3R× patient group, no statistically significant dose (*D*_max_ and *D*_mean_) differences (*p* > 0.2 from the paired Student *t*‐test) were found for both left and right cochlea. For 2R× patient group, no dose differences were observed for left cochlea, while the TSP had statistically significant dose reduction compared to the CP for right cochlea.

Table 2 lists the average and standard deviations of *D*_max_ for all serial organs for both 2R× and 3R× patient groups, as well as the *p*‐value from the paired Student *t*‐test. Compared to the CP, KBP had statistically significant reduction of the maximum dose (*p* < 0.05) to the mandible and spinal cord, and no significant maximum dose differences were observed for brainstem and brachial plexus. Between the CP and TSP, the only statistically significant differences we observed was maximum dose to the spinal cord.

**TABLE 2:** Maximum dose comparison between the CP, KBP and TSP for serial organs

		Mandible	Spinal cord	Brainstem	Brachial plexus
2Rx	CP	64.3 ± 5.3	40.8 ± 9.7	39.5 ± 11.4	60.1 ± 4.1
	KBP	61.4 ± 6.9	38.3 ± 8.5	37.8 ± 12.4	59.8 ± 2.9
	*p*	<0.01	<0.01	0.18	0.58
	TSP	63.4 ± 5.3	38.4 ± 9.0	38.0 ± 12.6	60.5 ± 3.1
	*P*	0.06	<0.01	0.09	0.36
3Rx	CP	62.7 ± 6.9	41.2 ± 3.9	34.1 ± 10.7	64.8 ± 0.9
	KBP	60.3 ± 8.3	37.7 ± 2.3	33.5 ± 13.1	64.1 ± 1.7
	*p*	<0.01	<0.01	0.58	0.05
	TSP	61.5 ± 7.3	38.2 ± 3.1	33.6 ± 11.7	64.3 ± 1.0
	*p*	0.05	<0.01	0.49	0.05

*Note*: Also lists *p*‐values from the paired Student *t*‐test.

The average and standard deviations of selected dosimetric indices for the parallel organs are listed in Table 3 for the CP, KBP, and TSP. In comparison with the CP, KBP had statistically significant dose reductions to all parallel organs for both the 2R× and 3R× patient groups. Except for left parotid gland (*D*_mean_ and *V*_30_ for both 2R× and 3R× patient groups) and oral cavity (*D*_mean_ for 3R× patient group), the TSP had statistically significant dose reductions for all parallel organs compare to the CP.

**TABLE 3:** Dosimetric comparison between the CP, KBP, and TSP for parallel organs

		Larynx *D*_Mean_	OralCavity *D*_Mean_	Pharynx *D*_Mean_	Left parotid *V*_50_	Right parotid *D*_Mean_	*V*_30_	*D*_Mean_	*V*_30_
2R×	CP	41.8 ± 15.9	34.4 ± 9.3	48.9 ± 11.2	54.3 ± 28.0	21.8 ± 11.4	29.3 ± 23.4	26.7 ± 13.1	36.8 ± 23.3
	KBP	34.8 ± 14.3	30.1 ± 5.7	39.9 ± 6.5	26.0 ± 15.5	17.6 ± 5.8	18.5 ± 11.7	21.0 ± 9.0	23.7 ± 16.8
	*p*	<0.01	<0.01	<0.01	<0.01	0.01	<0.01	<0.01	<0.01
	TSP	37.8 ± 15.9	32.1 ± 9.3	46.3 ± 19.7	46.5 ± 23.5	21.5 ± 9.2	27.1 ± 17.0	25.5 ± 11.5	33.0 ± 20.2
	*p*	< 0.01	0.02	< 0.01	< 0.01	0.76	0.38	0.04	0.02
3R×	CP	56.7 ± 18.0	37.7 ± 15.4	51.0 ± 18.0	60.7 ± 35.3	24.1 ± 5.5	31.5 ± 10.0	25.8 ± 6.9	35.0 ± 12.6
	KBP	49.3 ± 13.7	34.9 ± 12.2	46.2 ± 16.7	47.2 ± 34.4	19.1 ± 5.3	19.8 ± 10.1	19.2 ± 4.0	20.7 ± 7.5
	*p*	< 0.01	0.03	< 0.01	< 0.01	< 0.01	< 0.01	< 0.01	< 0.01
	TSP	54.1 ± 13.1	37.6 ± 14.9	49.9 ± 17.9	55.6 ± 36.1	23.0 ± 6.2	29.4 ± 11.8	23.9 ± 5.8	31.2 ± 10.8
	*p*	0.02	0.89	0.04	0.03	0.06	0.20	0.01	0.02

*Note*: Also lists *p*‐values from the paired Student *t*‐test.

**FIGURE 5:**
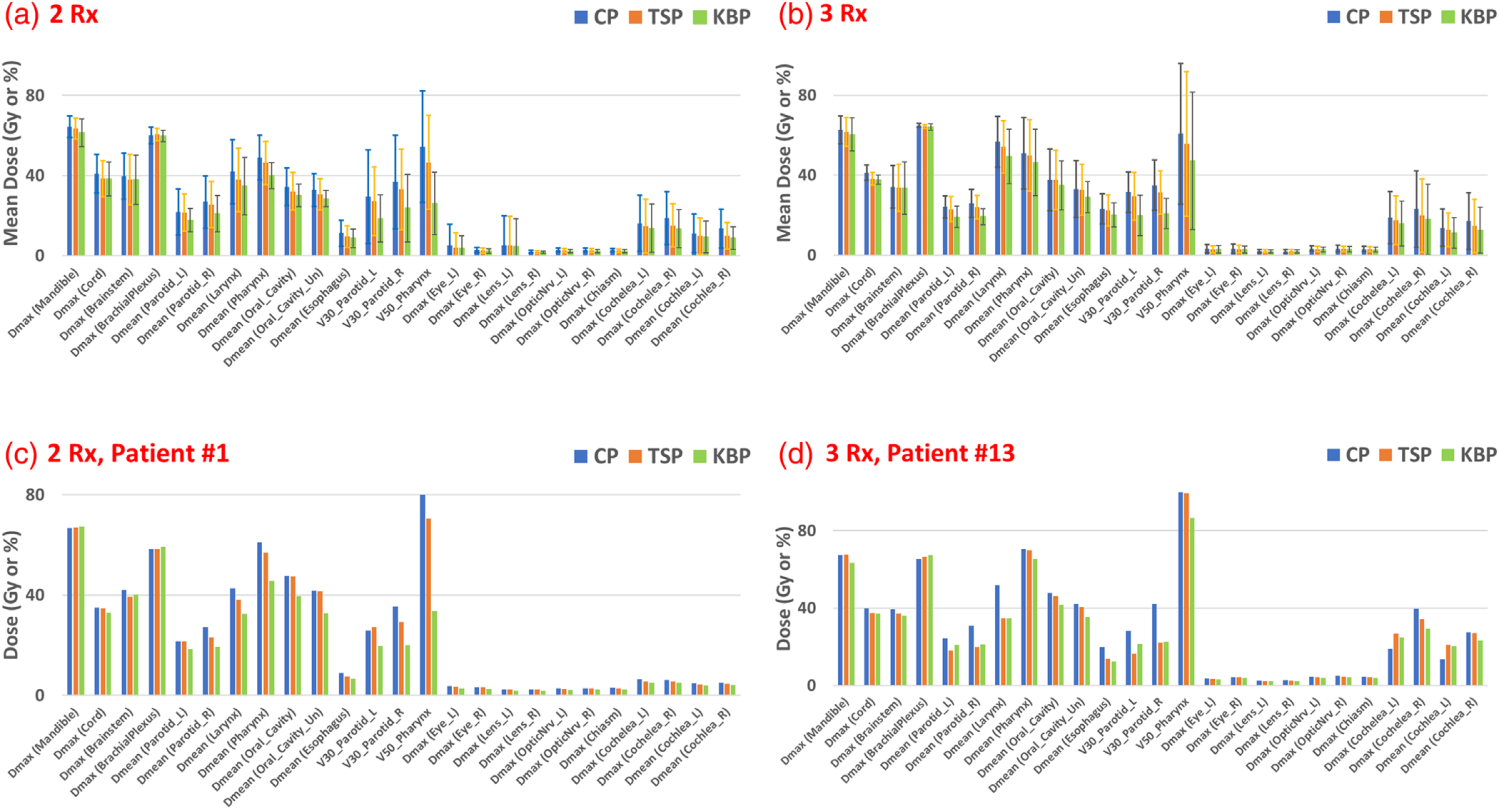
Dosimtric comparison for critical organs between the CP and TSP. Mean and variance dose metrics for (a) 2R× patient group and (b) 3R× patient group. Dose metrics for two representative patients for (c) 2R× patient group and (d) 3R× patient group

Figure 5 shows side‐by‐side comparison of the mean and variance of dosimetric indices among the CP, KBP and TSP of all critical organs for (A) 2R× patient group and (B) 3R× patient group. As shown in Figure 5a,b, compared with step 1 optimization (KBP), the OAR sparing gets a little worse after the two‐step approach (TSP) because of the improved target coverage in the second step. As a demonstration, the dosimetric comparison of the CP, KBP and TSP is also shown for two representative patients from different patient group in Figure 5c,d.

**FIGURE 6:**
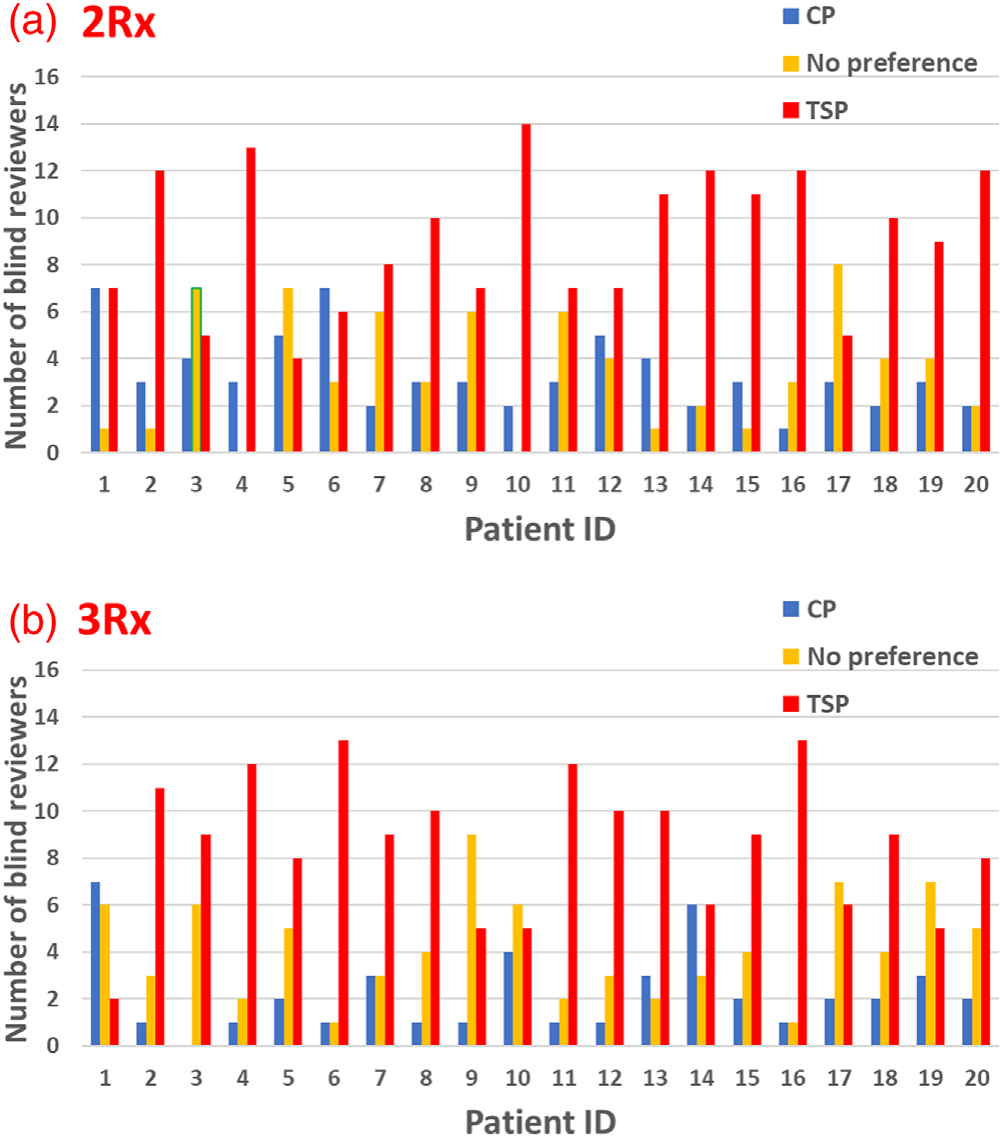
Bar graph showing the preferences between the two plans on blind review. Reviewers were given the option to choose CP, TSP or no preference

For all patients in this study, all reviewers agreed that the TSP and CP were clinically acceptable for treatment. As summarized in Table 4, the blind review results showed that 78.9%/85.3% (2R×/3R×) of the TSP were either “preferred” or marked as “no preference” compared with the CP. When removing “no preference” responses 57.2%/57.5% (2R×/3R×) of the TSP were selected as the preferred choice. Figure 6 shows the breakdown of the blind review result on a per patient level. 100% of TSP were preferred or had no preference for all 40 patients studied. If we removed “no preference” responses, there were only three patients (#5, #6 from 2R× and #1 from 3R×) where the CP choice exceed the TSP choice by all reviewers.

**TABLE 4:** Percentage of patients received the preference votes

	CP	TSP	No preference	RP + No preference
2R×	21.1%	57.2%	21.7%	78.9%
3R×	14.7%	57.5%	27.8%	85.3%

## 4 DISCUSSIONS

One of the biggest challenges in HN planning is that plan complexity increases with the number of targets and OARs involved and has a strong dependence on the size and location of the targets. The final achieved plan quality using traditional planning techniques is highly subjective and remains dependent on the knowledge and experience of the planners creating the potential for wide variation across institutions. Automation of treatment planning is rapidly developing and has the potential to increase planning efficiency while producing consistent and clinically acceptable treatment plans. Much effort has been given to developing automated solutions with some success for certain diseases in HN planning, the practical applications in the clinic are still limited and institution dependent.

In this study we developed a new two‐step optimization algorithm for HN treatment. A knowledge‐based planning solution (RapidPlan model) was incorporated in step 1 of the optimization. After some trial and error, we obtained better results by using un‐involved parallel organs (larynx, pharynx, oral cavity and parotid glands) in the RapidPlan model building. Furthermore, a posterior avoidance helper structure was also built into the model to improve normal tissue sparing. We believe one key principle of our new two‐step method is optimizing the OAR and normal tissue sparing in the first optimization step. Target coverage is accomplished in the step 2 of the optimization, as well as the control of the PTV hot spot, maximum dose to all serial organs, and improvement of plan conformity with the help of ring structures. Step 2 optimization starts with the previous step 1 optimization result, and then manually go back to MR level 3. By setting that, we limit larger MLC movements which are available during the earlier levels, which maintain the OAR and normal tissue sparing achieved in step‐1 optimization.

We tested the TSP algorithm by comparing 40 patients with two different prescription settings. The TSP were able to achieve all planning DVH constrains for serial organs, and provide comparable, if not better, doses to the parallel organ when compared to the CP (Tables 2 and 3). For the majority of patients studied, the TSP provided slightly better, or similar conformity, for the high‐dose PTV, and better conformity for the low‐dose/intermediate‐dose PTVs and 45 Gy isodose lines compared to the CP (Figure 2).

Very promisingly all reviewers agreed that the new TSP produced clinical acceptable plans for all 40 patients. Blind review results showed that approximately 80% (78.9% for 2R× group and 85.3% for 3R× group) of the TSP were either “preferred” or “no preference” compared to the CP. Additionally, when evaluating the results of each patient individually, 100% of the TSP received “preferred” or “no preference” by a majority of the respondents. We interpret this as a success as the TSP would be used clinically and by deemed at least as good or better than the CP by a majority of those studied. Even when removing “no preference” responses, there were only three patients (#5, #6 from 2R×, and #1 from 3Rx) where the CP choice exceed the TSP choice (Figure 6). Comments from the blind reviewers as to why they chose the CP over TSP for those three patients include: slightly more conformal, slightly better midline sparing, reduced dose to oral cavity and teeth. The RapidPlan model was built with 3R× plans, thus, we would expect the blind review result for the 3R× patient group to possibly outperform the 2R× patient group.

Currently the new TSP still needs a planner's input at different stages in the planning process. Our long‐term goal is to automate the entire planning process as much as possible. To meet this goal, we have developed several tools to aid in preparation of treatment planning. First, an atlas‐based segmentation tool (MIM Software, Cleveland, OH) has been developed to generate all OARs for physician review and modification. Second, automated workflow (MIM Software) has been developed to create planning PTVs from original human‐drawn PTVs, un‐involved structures, and helper structures (ring and post‐avoidance) for planning purpose. Other ongoing efforts include implementation of Eclipse scripting tools to auto‐generate the treatment plan beams, prescription, load the RapidPlan model, match all the structures in RapidPlan, modify target and OAR dose constrains and weightings, etc. We want to acknowledge that currently no automated planning solutions can eliminate the need for human judgement and evaluation. The planner should make the final decision whether the target coverage and OAR sparing is reasonable based on an individual patient's anatomy. Additional optimization may be necessary for some patients based on physician feedback.

Despite there being quite a few publications highlighting the success of automated planning over traditional planning, the implementation of automated planning into the clinical practice is remains challenging. The typical time planners spend on a HN plan by using traditional planning technique at our clinic ranges from 2.5 h to 6 h, depending on the planner's experience and the complexity of the plan. For the new TSP, typical time spend on the first step is about 20–25 min (beam arrangements, RapidPlan model execution). We want to point out that the only time planner required to interact with the planning process at step 1 is to pause the optimization at MR level 1 for 5–10 min. Typical time spend on step 2 optimization is ranged from 20 min to 40 min, depending on plan complexity. We were able to achieve good plan quality by one round of step 2 optimization for majority of patients in this study. Another round of step 2 optimization was needed for some patients. We believe the extra time spend on the planning in the traditional strategy is due to multiple rounds of trial‐and‐error since planners do not know in advance what level of OARs sparing they can achieve for a new patient. In the TSP, step 1 (RapidPlan approach) provides a good reference of OARs sparing based on historical clinical data, what the planners need to do is try to improve the target coverage and plan conformity while try to maintain the OAR sparing achieved in step 1.

For all 40 patients in this study, we were able to achieve reasonable plan quality about 60–90 min by using the new TSP. At our institution, the TSP has been implemented into routine clinical practice since January 2022 with more than 20 HN patients being planned and approved by physicians for treatment.

## 5 CONCLUSIONS

Our novel TSP allows plans to be created within 90 min time frame while offering significant improvements in plan quality and less inter‐planner variability as compared to traditional planning.

## AUTHOR CONTRIBUTION

Han Liu: designed the analysis, collected the data, performed the analysis and drafted the manuscript. Benjamin Sintay: assisted with the study design and drafted the manuscript. David Wiant: assisted with the study design and drafted the manuscript.

## CONFLICT OF INTEREST

This research was supported by a grant from Varian Medical Systems, Palo Alto, CA.
